# *Nocardia* infection: a rare case report of a cerebellar mass

**DOI:** 10.3389/fmed.2025.1687102

**Published:** 2025-09-29

**Authors:** Izzuddin Azaharuddin, Mohammad Nizam Mokhtar, Chuan Hun Ding, Mohd Imree Azmi, Farah Hanim Abdullah, Yik Peng Su, Affiza Zainudin

**Affiliations:** ^1^Department of Anaesthesiology and Intensive Care, Faculty of Medicine, Univerisiti Kebangsaan Malaysia, Kuala Lumpur, Malaysia; ^2^Department of Medical Microbiology and Immunology, Faculty of Medicine, Universiti Kebangsaan Malaysia, Kuala Lumpur, Malaysia; ^3^Department of Radiology, Faculty of Medicine, Universiti Kebangsaan Malaysia, Kuala Lumpur, Malaysia

**Keywords:** *Nocardia*, infection, central nervous system, cerebellar mass, intensive care unit

## Abstract

**Introduction:**

Central nervous system (CNS) nocardiosis is a rare but lethal opportunistic infection, presenting a formidable diagnostic and therapeutic challenge, especially in immunocompromised populations. The most common presentation is a cerebral abscess.

**Case presentation:**

We report the case of a 53-year-old male with a history of relapsed and refractory multiple myeloma who presented with an acute change in mental status and ataxia. Initial clinical suspicion centered on a posterior circulation ischemic event. However, magnetic resonance imaging (MRI) revealed a large, rim-enhancing cerebellar mass with associated obstructive hydrocephalus. The patient underwent surgical excision of the lesion, and subsequent microbiological culture confirmed an abscess caused by a *Nocardia* species. He was treated with a prolonged course of targeted antimicrobial therapy, including intravenous imipenem, followed by oral linezolid and trimethoprim-sulfamethoxazole. At the 3-month outpatient follow-up after discharge, the patient was found to have thrombocytopenia, attributed to the side effects of oral trimethoprim-sulfamethoxazole. Treatment was subsequently maintained with oral linezolid 600 mg for 1 year. Despite improvement of the abscesses, the patient ultimately succumbed to the progression of his long-standing multiple myeloma.

**Conclusion:**

This case underscores the importance of including uncommon opportunistic pathogens like *Nocardia* in the differential diagnosis of CNS lesions in immunocompromised patients. A definitive diagnosis via microbiological analysis of tissue specimens is imperative to guide appropriate antimicrobial therapy and achieve a favourable clinical outcome.

## Introduction

The genus *Nocardia* comprises a diverse group of aerobic, gram-positive, weakly acid-fast bacilli that form characteristic branching filaments (1). These organisms are ubiquitous in the environment but are an infrequent cause of human disease. The taxonomy of the genus is complex and continuously evolving, with numerous species now recognized as clinically significant, which complicates identification efforts (2, 3). When nocardiosis occurs, it can manifest as a severe localized or disseminated process. The primary route of acquisition is typically via inhalation, leading to pulmonary disease with potential for subsequent hematogenous dissemination to other organ systems (4, 5). The central nervous system CNS is the most common site of extrapulmonary disease, affecting a significant proportion of patients with systemic nocardiosis (6, 7).

The diagnosis of CNS nocardiosis is often challenging due to its nonspecific clinical and radiological features, which can mimic other pathologies such as infectious etiologies, or more notably, metastatic brain tumors (6, 8). Immunocompromised individuals represent the highest-risk population, with corticosteroid use being a common predisposing factor (7, 9). This highlights the need for a high index of suspicion in this vulnerable patient group.

We present a compelling case of a patient with a history of multiple myeloma who developed a *Nocardia* cerebellar abscess. This case highlights the diagnostic difficulties of such a presentation and emphasizes the necessity of considering rare opportunistic pathogens in patients with a compromised immune system.

## Case presentation

A 53-year-old male with a five-year history of relapsed and refractory immunoglobulin A kappa (IgAκ) multiple myeloma presented with an acute change in mental status and profound instability, specifically altered consciousness and ataxia with no other cerebellar signs. The patient was also noted to be semi-dependent in his activities of daily living. His medical history was notable for multiple lines of therapy for the kappa (IgAκ) multiple myeloma, including proteasome inhibitors, immunomodulatory drugs, and an autologous stem cell transplant. His course had been complicated by numerous sequelae, including pathological fractures, hypercalcemia-induced delirium, acute kidney injury, and neutropenic sepsis. At the time of presentation, he was receiving a salvage regimen of daratumumab, pomalidomide, and dexamethasone.

**Figure 1:**
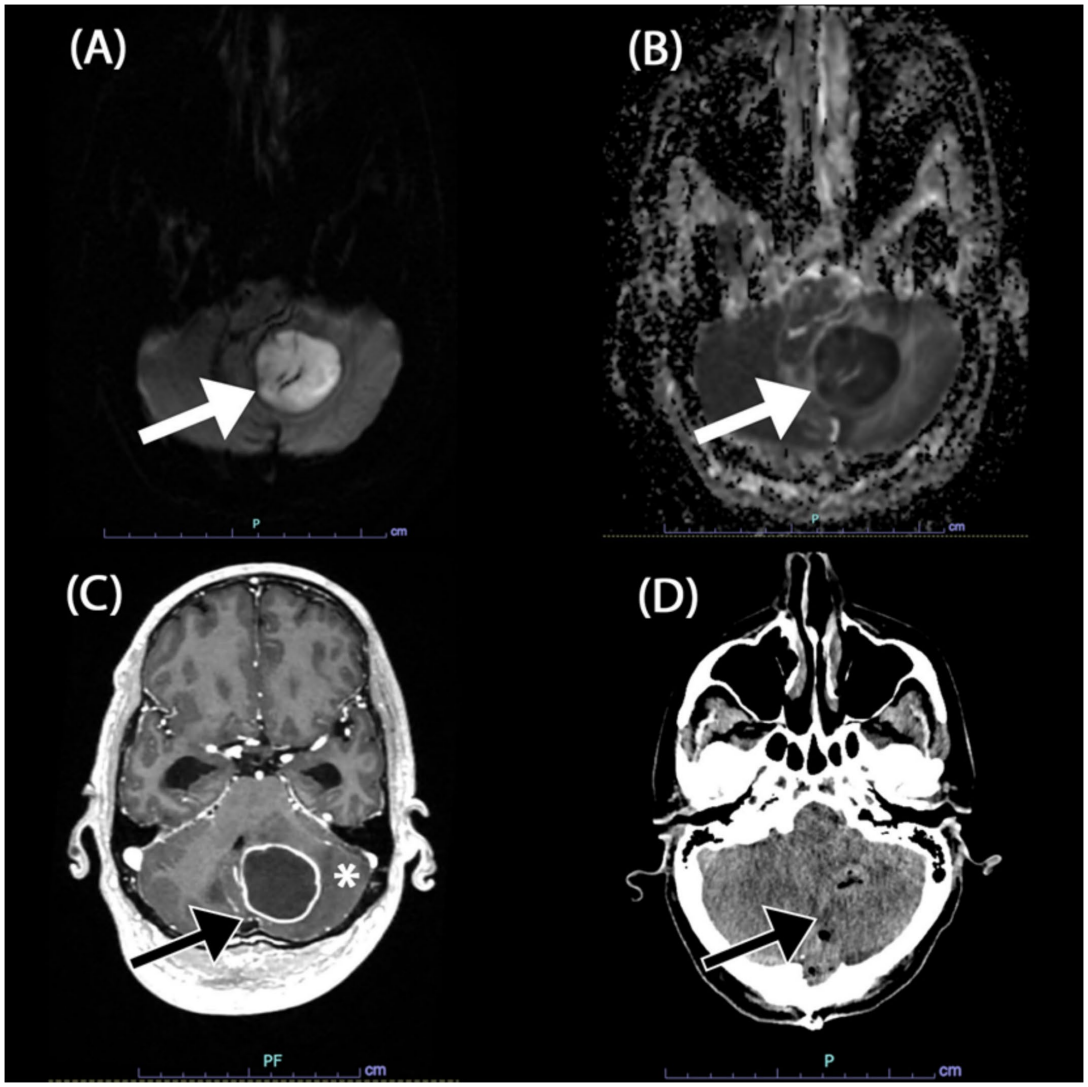
Magnetic resonant imaging (MRI) of the brain in axial views. **(A,B)** DWI b1000 and ADC showed restricted diffusion of the mass in the left cerebellum (white arrow). **(C)** Post-gadolinium contrast T1-weigted image revealed rim enhancing component of the mass (black arrow) associated with surrounding oedema (*). Note the compression onto the fourth ventricle causing obstructive hydrocephalus (not shown). **(D)** Post-operative non-contrasted computed tomography (CT) of the brain in axial view revealed the post operative air locules (black arrow) in the left cerebellum without bleed and resolution of fourth ventricle compression.

The initial symptoms of altered consciousness and ataxia were attributed to a presumed posterior circulation ischemic event. A magnetic resonance imaging (MRI) revealed a large, 3.4 × 3.5 × 3.2 cm mass in the left cerebellum (Figures 1A–C), which exhibited restricted diffusion and rim enhancement. The mass was exerting a significant effect on the fourth ventricle, resulting in obstructive hydrocephalus. The leading differential diagnoses at this juncture were CNS lymphoma and a cerebellar abscess. Diffusion-weighted imaging (DWI) was also performed (Figures 1A,B) and showed findings that were most suggestive of an abscess.

**Figure 2:**
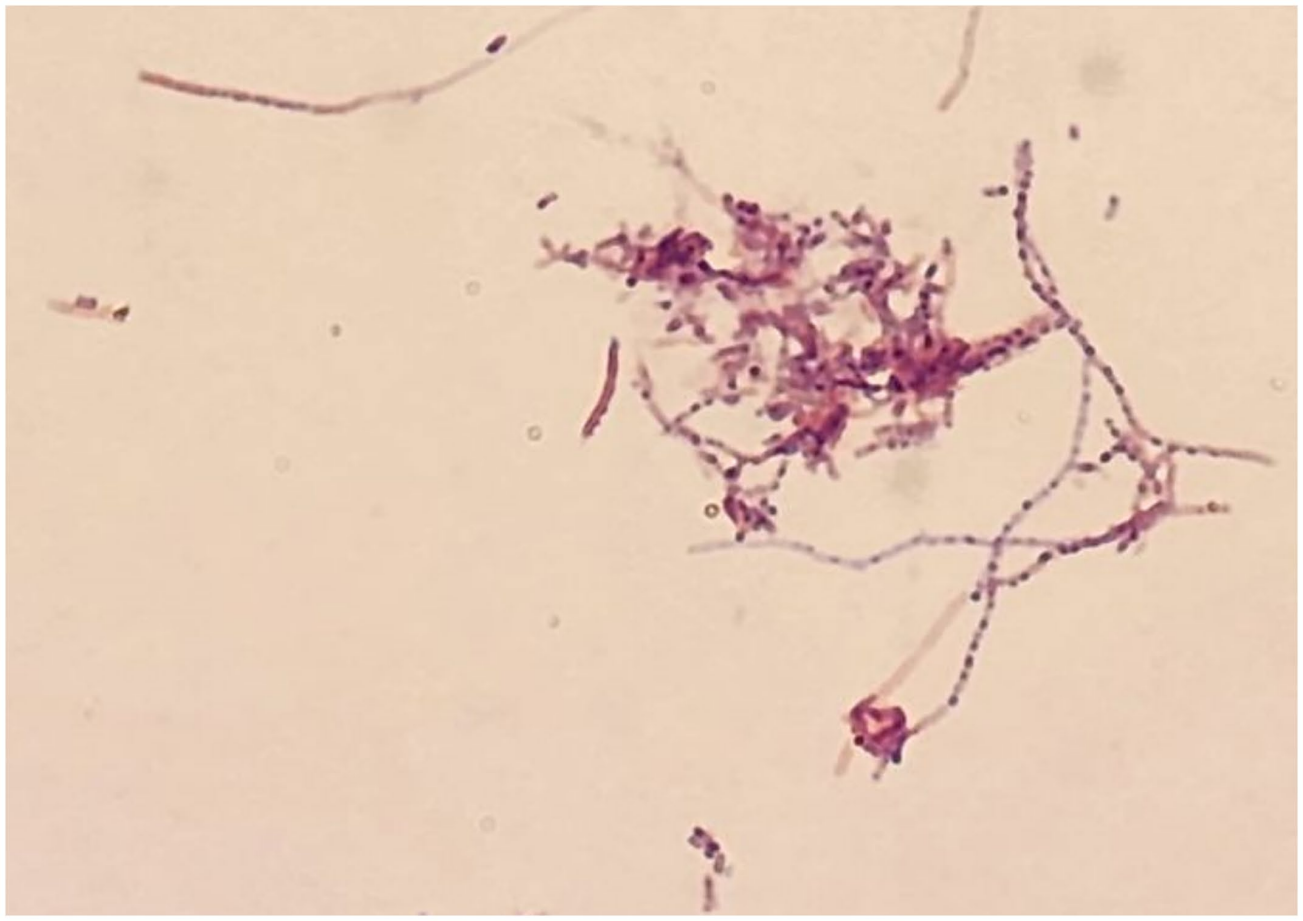
Microscopic examination of the excised cerebellar lesion showing branching, filamentous Gram-positive bacteria consistent with *Nocardia* species (Gram stain, 1,000× magnification).

**Figure 3:**
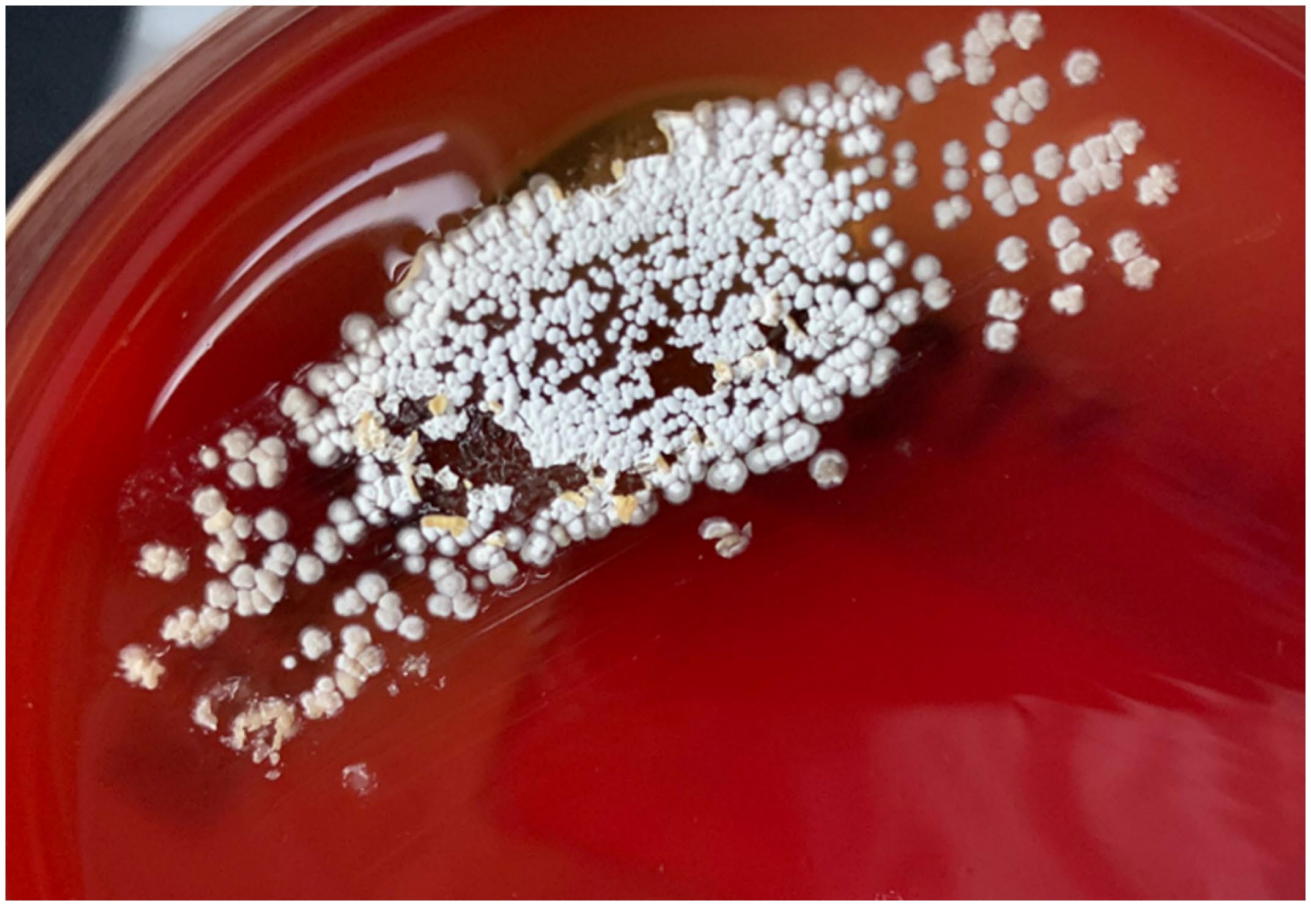
Culture of the excised lesion on blood agar demonstrating the characteristic dry, chalky, white-tan colonies of *Nocardia* species.

A right-sided external ventricular drain (EVD) was placed to treat the obstructive hydrocephalus. Cerebrospinal fluid (CSF) analysis revealed a high opening pressure but was otherwise unremarkable, with low protein (<68 mg/L), elevated glucose (5.04 mmol/L), and negative cultures. Forty-eight hours after EVD placement, the patient underwent surgical excision of the cerebellar lesion. Intraoperatively, the mass was noted to be encapsulated and rubbery, containing a yellowish, caseous material. A definitive diagnosis was established by the microbiology laboratory, where cultures of the abscess material yielded *Nocardia* species (Figures 2, 3). The isolate demonstrated sensitivity to imipenem and trimethoprim-sulfamethoxazole (cotrimoxazole).

The patient’s postoperative course was managed in the intensive care unit (ICU). An extensive evaluation for other concurrent infections, including serial blood cultures and a comprehensive panel of serologies, was negative. A non-contrasted CT brain did not show any significant bleed and resolved obstructive hydrocephalus (Figure 1D). A CT scan of the chest, abdomen, and pelvis revealed findings consistent with ventilator-associated pneumonia but no definitive primary focus of nocardiosis.

His antimicrobial regimen was initiated with intravenous imipenem 500 mg four times daily for 28 days and intravenous trimethoprim-sulfamethoxazole for 13 days. He was subsequently transitioned to a long-term oral regimen of oral linezolid 600 mg twice daily and oral trimethoprim-sulfamethoxazole two tablets a day twice weekly for a total duration of 1 year.

Over the ensuing weeks, the patient exhibited a steady and significant neurological recovery. His level of consciousness, coordination, and speech gradually returned to his functional baseline. A follow-up CECT brain one-month post-surgery demonstrated a marked reduction in the size of the cerebellar lesion at 1.5 × 2.4 × 2.4 cm (previously 3.4 × 3.5 × 3.2 cm) with a lesser degree of mass effect. He was ultimately discharged in a stable condition to complete his extended course of antibiotic therapy as an outpatient.

**Figure 4:**
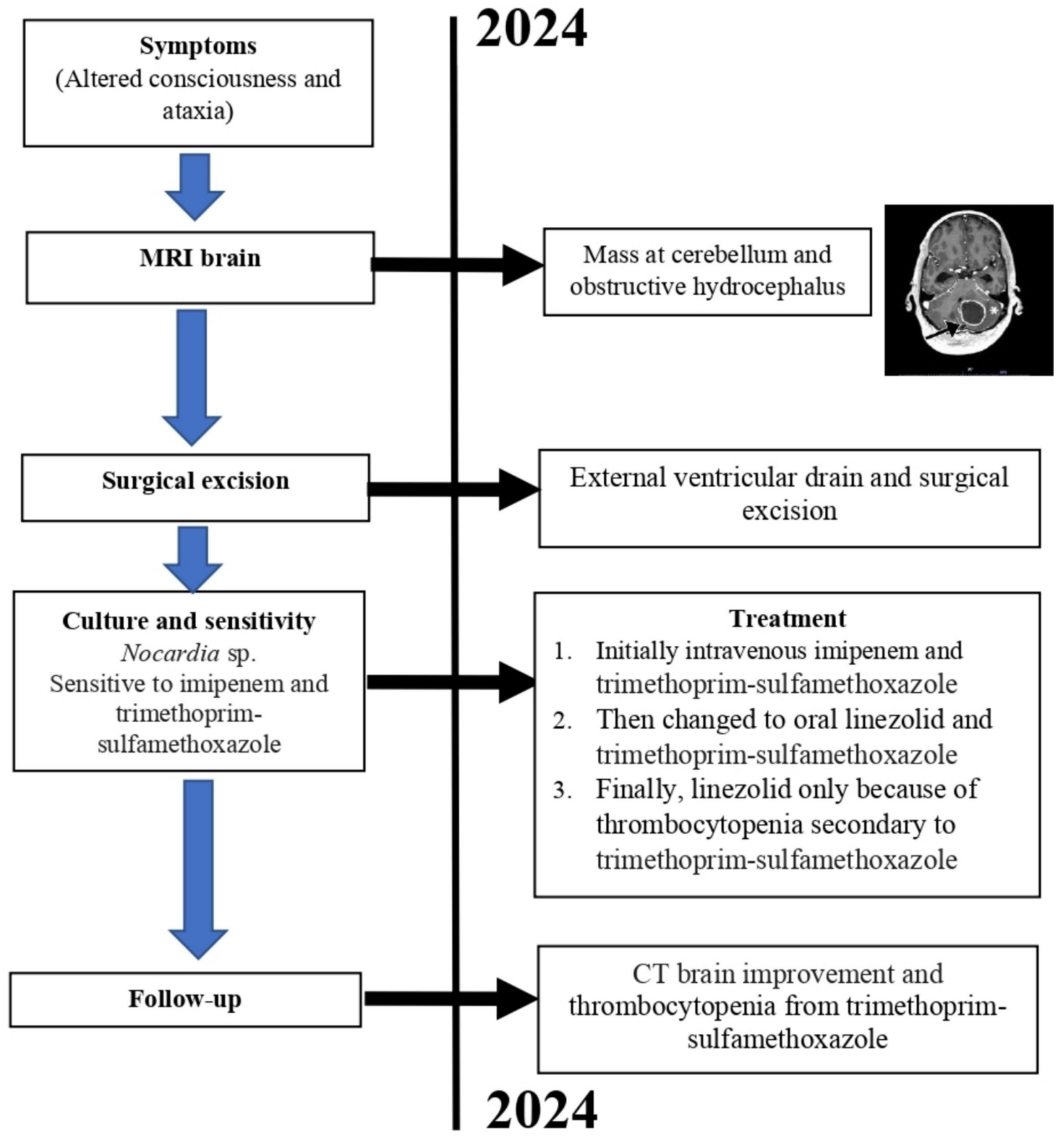
Case report timeline. Presented according to CARE guidelines.

During the outpatient clinic visits after 3 month being discharged, patient was found to have thrombocytopenia and was attributed to a side effect of oral trimethoprim-sulfamethoxazole and the treatment was only maintained with oral linezolid 600 mg for one-year duration. Patient condition of consciousness, coordination, and speech has significant improvement. Six months after the procedure, patient developed recurrence of multiple myeloma but opted for palliative care and refused reinduction of chemotherapy. Despite improvement of the abscesses, the patient succumbed due to progression of his long-standing multiple myeloma. The clinical timeline of the patient progression in shown in Figure 4.

## Discussion

This case provides a compelling illustration of a rare but critical opportunistic infection in an immunocompromised patient. The patient’s underlying multiple myeloma and extensive treatment history created a state of profound T-cell-mediated immune deficiency, which is the quintessential risk profile for invasive nocardiosis, particularly with the use of newer agents (10, 11). The pathogenesis of nocardial dissemination to the CNS is facilitated by specific virulence factors, which allow the organism to evade phagocytic killing and promote hematogenous seeding of the brain (4).

The diagnostic journey in this case highlights several classic challenges of CNS nocardiosis. The initial misdiagnosis of a stroke is a common pitfall, driven by the nonspecific and often insidious onset of neurological symptoms, which can lead to critical delays in appropriate treatment (7). Furthermore, the neuroimaging finding of a ring-enhancing lesion, while suggestive of an abscess, is not pathognomonic and shares features with high-grade neoplasms and other infections, making a definitive radiological diagnosis challenging (6, 8). The patient’s clinical presentation, combined with the presence of multiple myeloma, placed CNS lymphoma high on the list of differential diagnoses. The definitive diagnosis was only established through surgical excision of the lesion, which confirmed the presence of a pyogenic abscess and, importantly, yielded cultures positive for *Nocardia* sp.

A crucial diagnostic principle underscored by this case is the low utility of CSF analysis for encapsulated brain abscesses. The negative CSF cultures are consistent with the literature, where the diagnostic yield of CSF is reported to be low in the absence of concomitant meningitis (7, 12, 13). This emphasizes that for a patient with a suspected nocardial abscess, a lumbar puncture is an insufficient diagnostic step, and clinical efforts must be aggressively directed toward obtaining a tissue specimen. Direct aspiration or excision of the lesion remains the gold standard for diagnosis, as it provides the highest yield for both histopathology and, critically, culture for antimicrobial susceptibility testing (7, 14). The extended time required for culture results creates a diagnostic-therapeutic gap where empirical therapy must be initiated without definitive susceptibility data.

The successful outcome in this patient was predicated on a multimodal therapeutic strategy that combined aggressive neurosurgical intervention with prolonged, targeted antimicrobial therapy (7, 14, 15). This combined approach is consistently associated with the best survival rates in CNS nocardiosis. Surgical intervention was vital not only for establishing the diagnosis but also for source control, reducing the intracranial mass effect, and decompressing the obstructed ventricular system.

The antimicrobial regimen was designed based on established principles for treating severe nocardiosis. The initial use of combination intravenous therapy with imipenem and trimethoprim-sulfamethoxazole is a standard approach, though more recent data suggest a trend toward using combinations with better CNS penetration (4, 7, 16). In this case, the regimen was complicated by the patient’s reaction to trimethoprim-sulfamethoxazole leading to thrombocytopenia, which necessitated a switch to oral Linezolid-based regimen only. This highlights the practical challenges of managing long-term antimicrobial therapy in a critically ill and immunocompromised patient. The transition to a long-term oral regimen containing linezolid is also consistent with current evidence. Linezolid offers excellent oral bioavailability and CNS penetration and has near-universal *in vitro* activity against *Nocardia* species, making it a cornerstone of modern therapy, particularly when prolonged treatment is required (4, 17, 18). The planned one-year duration of therapy is appropriate for CNS disease in an immunocompromised host, as shorter courses are associated with a higher risk of relapse (7). This extended duration, however, necessitates vigilant monitoring for potential toxicities, particularly the myelosuppression and neuropathy associated with long-term linezolid use (19, 20).

Although the abscess resulting from nocardiasis was managed successfully with the antibiotic regimen, the progression of his multiple myeloma prevented us from completing the planned one-year course of treatment.

## Conclusion

CNS nocardiosis remains a significant diagnostic and therapeutic challenge that requires a high index of suspicion, particularly in patients with profound immunosuppression. This case highlights the classic pitfalls in diagnosis, including non-specific clinical and radiological presentations, and reinforces the principle that a definitive diagnosis requires direct tissue sampling. An optimal outcome is contingent upon an aggressive, multidisciplinary approach that combines prompt neurosurgical intervention for source control with a prolonged course of targeted, multi-drug antimicrobial therapy guided by susceptibility testing and any adverse reaction related to the treatment.

## Data Availability

The raw data supporting the conclusions of this article will be made available by the authors, without undue reservation.
